# Society Meetings

**Published:** 1913-02

**Authors:** 


### SOCIETY MEETINGS.

The Eighth District Dental Society, President Dr. Walter H. Ellis, and The Buffalo Dental Association, President, Dr. Wesley M. Backus, held a joint dinner meeting at the Hotel Statler, Buffalo, January 11. Dr. Charles W. Bayliss, of Oneonta, President of the State Dental Society, spoke briefly, addressing his remarks especially to the younger members. John W. Van Allen, Esq., Chairman of the Industries Bureau of the Buffalo Chamber of Commerce, read a paper on Investments for the Professional Man. As physicians as well as dentists have been the prey of blue sky promotors, this paper will be printed in a subsequent issue. Dr. Arthur F. Isham showed stereopticon views of his trip to Japan last year.

The Seventeenth International Congress of Medicine will be held in London, August 5-12, 1913. Dr. Foveau de Courmelles of Paris, will report on the X-Rays and Radium in Gynaecology, and requests the profession to send him observations as soon as possible, addressing to 26 rue de Chauteaudun, Paris..

The Medical and Surgical League held a dinner meeting at the Hotel Statler, Buffalo, January 9. Dr. Frank Kleckner gave a paper on "The Tonsils, Their Hypertrophy and a Method of Enucleation." Drs. Wurtz and Michel were elected to membership.

The Buffalo Academy of Medicine has inaugurated the plan of subscription dinners, at moderate price, open to all members, for the entertainment of out-of-town speakers. The first dinner was held at the Hotel Statler, January 28, Dr. John A. Amyot, Director of the Laboratories of the Ontario Board of Health of Toronto, being the guest of honor. This is a reform for which we have personally pleaded for years. The original plan of making appropriations from the society funds for more or less private and necessarily limited entertainment was obviously objectionable. The resulting custom of placing the expense of entertainment upon certain officers has also been embarrassing in many ways, especially in that it has not been possible to arrange in an entirely satisfactory way for the meeting with the guest of those personally acquainted with him or professionally most desirous of the meeting. The present plan is fair to all, is from the guest's standpoint most gratifying—or at least the editor has personally liked that form of entertainment best when he has been the guest of out-of-town societies—and, most important of all, it does away with what in this country and in any profession, is a most regrettable tendency toward a cleavage into gentry and peasantry.

THE ELMIRA ACADEMY OF MEDICINE was organized in 1852. Meetings are held on the first Wednesday of each month, except July and August, the annual meeting being held in December. The President is Dr. Alexander Mark, the Secretary, Dr. Charles Haase, our Associate Editor. The membership consists of 63 active and 6 honorary fellows. On January 8, Dr. C. G. R. Jennings read a paper on "Apocynum"—a valuable though neglected drug—and Dr. E. T. Bush on "Post Graduate Work in N. Y. City."

---

THE HOSPITAL MEDICAL SOCIETY of Rochester held meetings January 2 and January 16. At the former, Dr. Ralph R. Fitch read on "Lateral Curvatures of the Spine: New Ideas." At the latter, Dr. Edmund C. Boddy read on "Medical Inspection in the Public Schools.

---

THE ROCHESTER PATHOLOGICAL SOCIETY met January 9, Dr. Frank F. Dow presenting a paper on "Conservatism."

---

THE ROCHESTER ACADEMY OF MEDICINE entertained Dr. Arthur D. Hirschfelder of Johns Hopkins University, at a dinner at the Rochester Club, January 8. Dr. Hirschfelder read a paper on "Some Clinical Features of Adams-Stokes Disease," illustrated by stereopticon. At the annual meeting at the Academy Home (a good name which is respectfully commended to other societies of similar nature—Ed.) officers were elected as follows: President, Charles E. Darrow, M. D.; Vice-Presidents, Lee A. Whitney, M. D., LeGrand A. Walker, M. D., Leighton R. Cornman, M. D., Clayton K. Haskell, M. D.; Secretary, Edward L. Hanes, M. D.; Treasurer, Wesley T. Mulligan, M. D.; Trustees, Edward B. Angell, M. D., Eugene H. Howard, M. D., Edward W. Mulligan, M. D.; Councillors, William B. Jones, M. D., Charles D. Young, M. D.; Member of Library Committee, Prof. Charles W. Dodge.

---

THE FEDERATION OF STATE MEDICAL BOARDS will hold its annual meeting at the Congress Hotel, Chicago, on Tuesday, February 15th, 1913.

Essayists, eminently qualified, will prepare papers upon the following subjects:

"Is Universal Reciprocity to be Desired?" "Should Medical Boards Require One or More Years of College Work Preliminary to the Study of Medicine?" "Should One or More Years in a Hospital be Required for Admission to the Examination for Medical Licensure?" "Rules and Regulations Governing Examinations for Medical Licensure." "Qualification of Examiners."

"What Fee Should be Required for the Examination?" "Benefit of Having a Single Federation of State Medical Boards and Method of State Board Record Keeping." "Means of Keeping Politics Out of State Board Affairs."

An invitation is extended to members of State Medical Examining and Licensing Boards, teachers in medical schools, colleges and universities, delegates to the Council on Medical Education of the A. M. A., to the Association of American Medical Colleges and all others interested in securing the best results in medical education and legislation.

The officers of the Federation are Arthur B. Brown, M. D., President, New Orleans; George H. Matson, M. D., Secretary-Treasurer, Columbus (State House), Ohio; James A. Duncan, M. D., Chairman Executive Committee, Toledo.

